# OKAIN: A comprehensive oncology knowledge base for the interpretation of clinically actionable alterations

**DOI:** 10.1515/med-2025-1289

**Published:** 2025-10-30

**Authors:** Zhenhua Yang, Chunwei Xu, Mingmin Wang, Xinxiu Meng, Kai Wang, Aodi Wang

**Affiliations:** Department of Medical, Shanghai OrigiMed Co, Ltd, Shanghai, 201114, People’s Republic of China; Hangzhou Institute of Medicine (HIM), Chinese Academy of Sciences, Hangzhou, Zhejiang, 310022, People’s Republic of China; School of Life Sciences, Fudan University, Shanghai, 200438, People’s Republic of China

**Keywords:** precision oncology, tumor knowledge base, genomic alterations, classification system, weighted evidence analysis

## Abstract

The increased use of next-generation sequencing in clinical genetic testing has resulted in the identification of several genetic variations with possible therapeutic implications. We developed OKAIN (https://szcube.origimed.com), an algorithm tool that assesses clinically actionable mutations using a precision oncology knowledge database. OKAIN employs a weighted evidence analysis system to deliver final clinical annotation outcomes for intricate variations. As of now, OKAIN has amassed over 100,000 variants in 1,239 cancer-associated genes, encompassing 12,409 entries of therapeutic evidence in 471 genes. This collection highlights 2,600 Level A evidence entries in 66 genes, with 864 entries derived from the National Medical Products Administration labels or Chinese guidelines. OKAIN acts as a precision oncology knowledge base for the assessment of clinically actionable alterations, integrating exhaustive data related to cancer-associated genomic variants and therapeutic efficacy. Analyzing patient variants with OKAIN reveals more actionable targeted therapy or immunotherapy options, potentially improving treatment outcomes.

## 1 Introduction

Precision oncology is a rapidly emerging field that involves the molecular profiling of tumors to discern diagnostic, prognostic, and therapeutic implications [1–3]. Patients are increasingly undergoing multigene sequencing of individual tumors to identify potential tumor biomarkers that could be effectively targeted with corresponding drugs. Both the Food and Drug Administration (FDA) and the National Medical Products Administration (NMPA) have approved numerous drugs based on these biomarkers, such as larotrectinib. For example, larotrectinib can be successfully used as a treatment when an NTRK fusion is identified in a solid tumor patient without a known acquired resistance mutation.

While there is a broad consensus on the interpretation of certain genomic alterations, such as epidermal growth factor receptor (*EGFR*) T790M and *EGFR* L858R, a large number of genetic variants that lack consensus for interpretation pose a significant challenge [4–6], including co-mutations and acquired drug-resistance variants. The use of precision medical technology in oncology has led to the widespread adoption of next-generation sequencing (NGS) large panel detection as the primary clinical approach. This method allows for comprehensive coverage of rare mutation targets, systematic identification of co-occurring mutations in tumors, and significant improvements in clinical detection efficiency. However, accurately interpreting rare mutations remains a major clinical challenge. Therefore, it is necessary to create a precision oncology knowledge base that offers centralized, freely accessible, and accurately interpreted clinical information on genomic data. This tool would assist clinicians in interpreting genomic alterations detected in patient tumor samples, enabling them to make optimal treatment decisions.

Existing knowledge bases designed to aid in the clinical interpretation of alterations include resources such as Clinical Interpretation of Variants in Cancer (CIViC) [4] and OncoKB [5]. However, most of these databases provide either the final clinical annotation results or multiple pieces of clinical interpretation evidence for the alterations. Additionally, thorough annotations of gene variations of Chinese drugs are not considered in most of those databases.

To address this issue, we present OKAIN, a knowledge base for precision oncology that assesses clinically actionable alterations. OKAIN includes a wide range of information related to genomic variants associated with cancer and their effectiveness in treatment. Its purpose is to offer physicians precise and structured interpretations of cancer mutations found in patients’ tumors, thereby facilitating clinical decision making.

OKAIN provides comprehensive insights into the therapeutic efficacy of co-mutations, acquired drug-resistant variants, and rare mutations, assisting physicians in precisely treating patients with complex variants. The knowledge base includes a weighted evidence analysis system that conducts a thorough analysis of the evidence related to the therapeutic efficacy of a tumor patient’s cancer-associated genomic variants. This system enables the acquisition of robust evidence of therapeutic efficacy in tumor patients.

## 2 Methods

**Figure 1:**
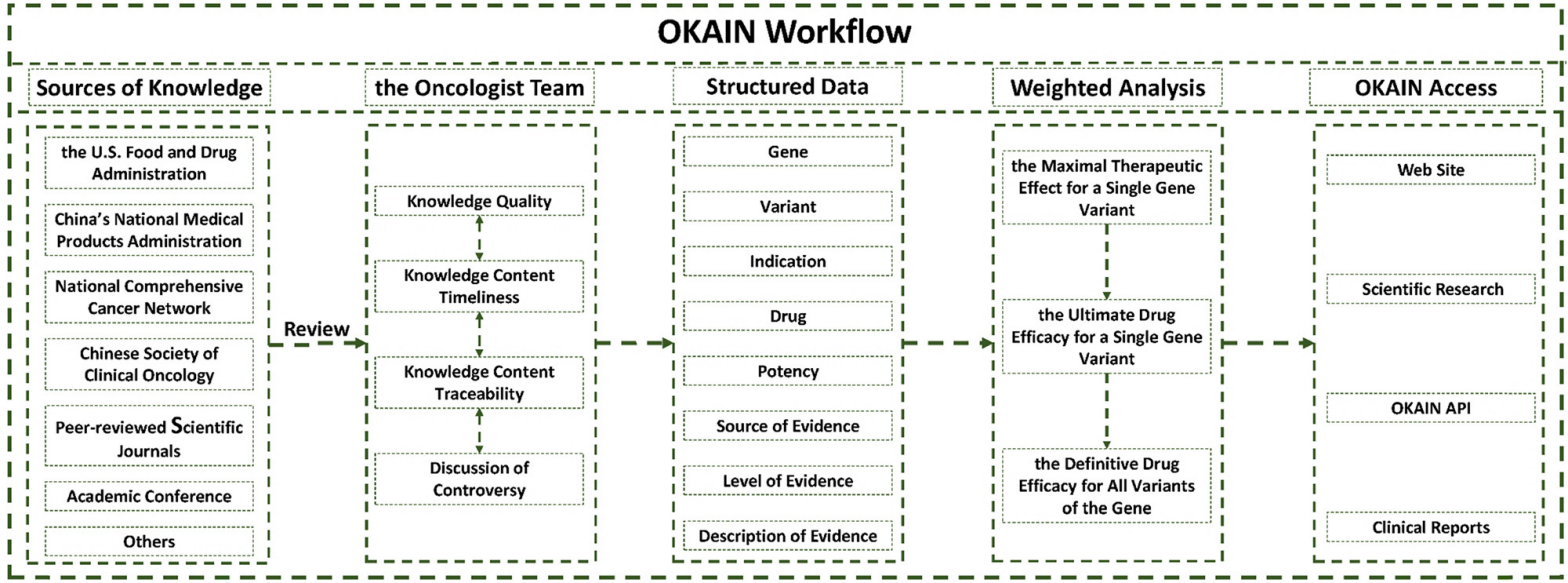
OKAIN workflow.

OKAIN (Version 1.0, 2024) accommodates clinically relevant cancer variants, including single nucleotide variants, frameshifts, insertions, deletions, gene rearrangements, copy number variations, and changes in expression levels. These variants are described unambiguously using the HGVS nomenclature [7,8]. OKAIN contains information about the biological effects and treatment implications of specific cancer genes and their variations. As shown in Figure 1, the data are sourced from a variety of authoritative entities, encompassing the FDA, NMPA, clinical guidelines, peer-reviewed scientific journals, and major conference proceedings, among others. Biological and clinical implications are meticulously reviewed by oncologist teams. To ensure a clear and precise representation of data, we will structure the information systematically. The structured data will include genes, variants, indication, drug, potency, source of evidence, level of evidence, and description of evidence. The weighted evidence analysis system to deliver final clinical annotation outcomes for intricate variations. The OKAIN dataset is mainly used for clinical research and to enhance OrigiMed’s clinical reporting. Users can access this dataset through an intuitive and interactive web interface at https://szcube.origimed.com.

In accordance with guidelines from the American Society of Clinical Oncology, the Association for Molecular Pathology, the College of American Pathologists, and the European Society for Medical Oncology [9,10], as well as information from public databases like OncoKB and consensus among oncologists, we have developed levels of evidence classification system (Table 1).

**Table 1:** Levels of evidence classification system

Potency	Category	Therapeutic
Response	Level A	A biomarker predictive of response to FDA/NMPA-approved therapies or those recommended by clinical guidelines in this indication
	Level B	A biomarker predictive of response to therapies, demonstrated by extensive retrospective studies or prospective investigations (Phase III clinical studies), in this indication
	Level C	C1. A biomarker predictive of response to FDA/NMPA-approved therapies or those recommended by clinical guidelines in another indication
		C2. A biomarker predictive of response to therapies, demonstrated by small-scale retrospective studies or early-stage prospective studies (Phase I or II clinical studies) in this indication
	Level D	D1. A biomarker predictive of response to therapies, demonstrated by a limited number of case reports in this indication
		D2. A biomarker predictive of response to therapies, supported by preclinical study results
Resistance	Level R1	A biomarker predictive of resistance to therapies, authorized by the FDA/NMPA or advised in professional clinical guidelines in this indication
	Level R2	R2B. A biomarker predictive of resistance to therapies, demonstrated by extensive retrospective studies or prospective investigations (Phase III clinical studies), in this indication
		R2C. A biomarker predictive of resistance to therapies, demonstrated by small-scale retrospective studies or early-stage prospective studies (Phase I or II clinical studies) in this indication
		R2D1. A biomarker predictive of resistance to therapies, demonstrated by a limited number of case reports in this indication
		R2D2. A biomarker predictive of resistance to therapies, supported by preclinical study results

**Figure 2:**
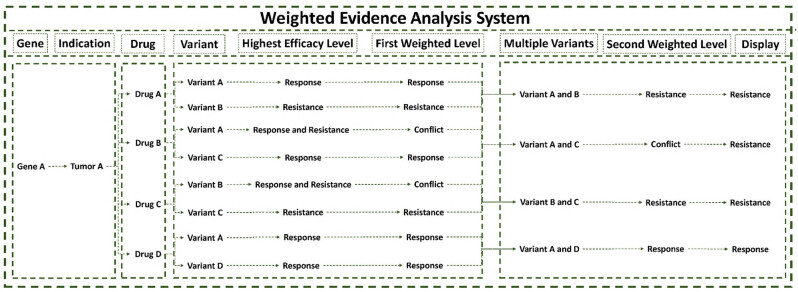
OKAIN’s weighted evidence analysis system. The Highest Efficacy Level indicates the maximal therapeutic effect for the specific genomic variant in response to a specific drug.

Continual research on the efficacy of genomic variants often produces conflicting evidence regarding the effectiveness of specific gene alterations. This poses a significant obstacle to the rapid interpretation of genomic alterations. To address this issue, OKAIN’s weighted evidence analysis system performs a comprehensive analysis of all therapeutic efficacy evidence for a specific genomic variant related to a particular drug, to determine the highest degree of efficacy for that drug. The highest level of efficacy is then evaluated against all variants of the gene, resulting in efficacy data for all gene variants in relation to the medication (Figure 2).

Within this framework, sensitivity evidence levels (A, B, C, D) exhibit direct correspondence with resistance evidence levels (R1, R2B, R2C, R2D), respectively, when weighted for a specific gene–tumor–drug–variant association. Sensitivity evidence levels follow a hierarchical order from highest to lowest (A → B → C → D), while resistance evidence levels descend correspondingly (R1 → R2B → R2C → R2D).

OKAIN prioritizes negative clinical evidence, discouraging the use of targeted therapies when the evidence suggests the therapy may be ineffective or controversial. When weighing the evidence level for multiple variants based on a specific drug, the evidence for drug resistance and conflicting evidence is prioritized over the level of evidence for sensitivity.

The Highest Efficacy Level represents the most clinically significant evidence tier for a specific gene–tumor–drug–variant, encompassing both sensitivity and resistance classifications following weighting analysis. For example, a variant may concurrently exhibit Level A, Level C, Level D, Level R2B, and Level R2D, wherein the Highest Efficacy Level would report Level A (highest sensitivity) and Level R2B (highest resistance).

The First Weighted Level denotes the definitive therapeutic efficacy prediction for a specific genomic variant in a given tumor type, derived directly from the Highest Efficacy Level. When conflicting evidence exists (e.g., concurrent sensitivity and resistance classifications), the system outputs “Conflict.”

The Second Weighted Level denotes the consensus therapeutic efficacy for all variants of a gene within a specified drug–tumor context, derived through aggregation of First Weighted Levels. This tier exclusively retains the highest efficacy level (sensitivity or resistance) observed across variants. When multiple distinct sensitivity types coexist within the definitive First Weighted Levels, prioritization follows the hierarchy: Resistance → Conflict → Response, preserving only the highest-priority classification. Should the Second Weighted Level manifest as “Conflict,” the final display will default to “Resistance” paired with the Highest Resistance Level, thereby prioritizing patient safety through avoidance of contentious therapeutic approaches.

## 3 Results

### 3.1 Levels of evidence

OKAIN categorizes the evidence for sensitivity to targeted therapies into four levels (A, B, C, and D) for each genetic alteration, based on the strength of clinical and preclinical data supporting the use of the mutation as a predictive biomarker.

**Figure 3:**
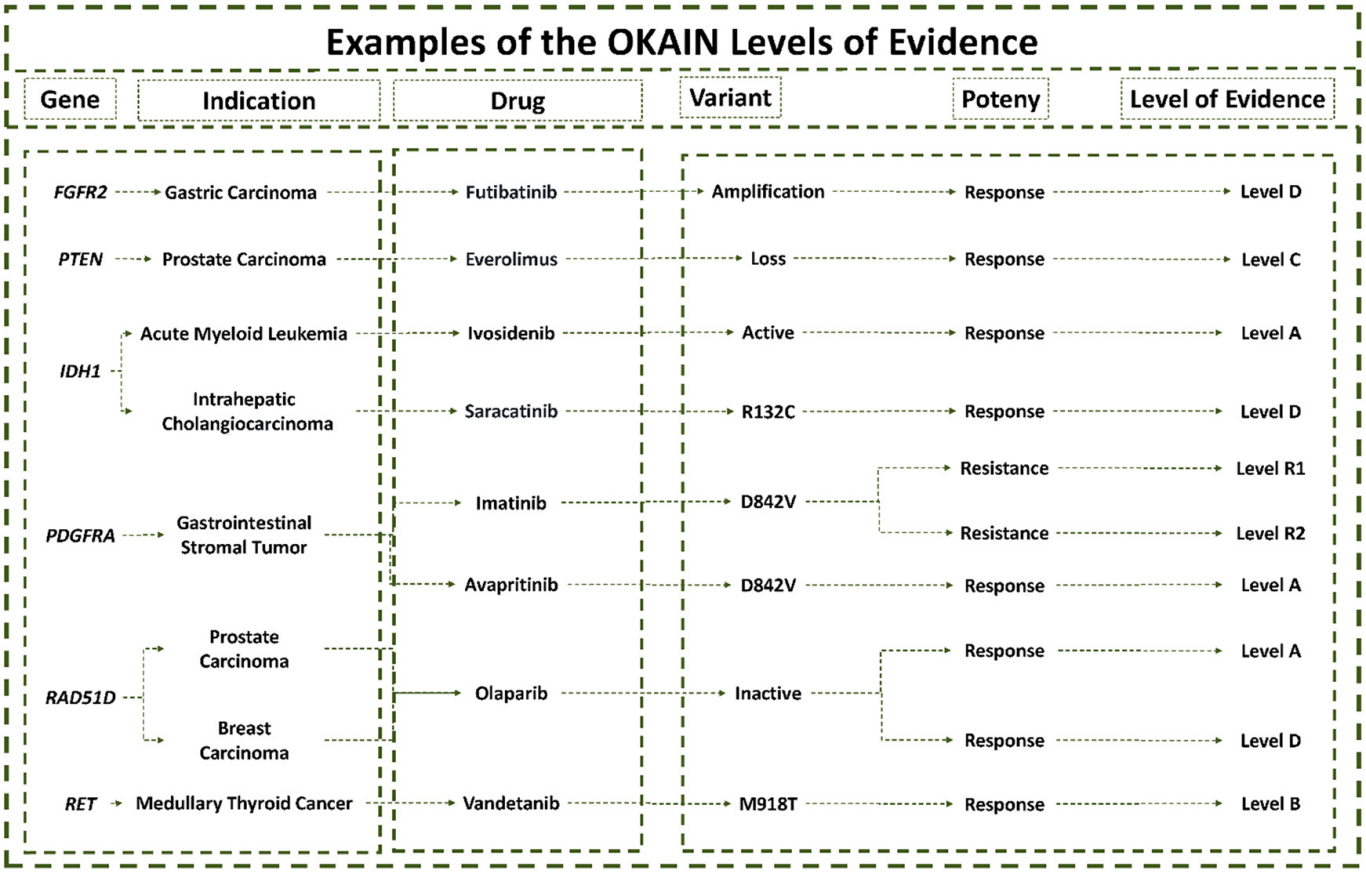
Examples of the OKAIN levels of evidence. The level of evidence shows the efficacy for a specific genomic variant related to a particular drug.

Level A includes specific alterations that have been approved by the FDA/NMPA or recommended by clinical professional guidelines as predictive of response to particular therapies in specific tumors. The FDA has approved avapritinib as a treatment for adult patients with unresectable or metastatic gastrointestinal stromal tumor (GIST) that harbor a platelet-derived growth factor receptor alpha (*PDGFRA*) exon 18 mutation, including *PDGFRA* D842V mutations. Avapritinib is a tyrosine kinase inhibitor that targets *PDGFRA* and *PDGFRA* D842 mutants, as well as multiple KIT proto-oncogene (*KIT*) exon 11, 11/17, and 17 mutations with half-maximal inhibitory concentrations (IC_50_s) less than 25 nM. *In vitro* cellular assays, avapritinib inhibited the autophosphorylation of *KIT* D816V and *PDGFRA* D842V mutants, which are associated with resistance to approved kinase inhibitors. The IC_50_ for *KIT* D816V was 4 nM and for *PDGFRA* D842V was 30 nM. Therefore, the use of avapritinib to treat GIST patients with *PDGFRA* D842V mutations is classified as Level A (Figure 3).

Level B includes specific alterations that serve as predictive biomarkers of response to therapies. These alterations have been established through large-scale retrospective studies or prospective studies, at least Phase III, in particular types of tumors. The ZETA Trial, a Phase III study, showed that patients with medullary thyroid cancer (MTC) who had a somatic *RET* M918T mutation had a higher objective response rate to vandetanib (55%) than patients with sporadic MTC tumors that lacked a somatic M918T mutation (33%) [11]. *In vitro* studies have shown that vandetanib inhibits the tyrosine kinase activity of various receptor tyrosine kinases, including the *EGFR* and vascular endothelial growth factor (*VEGFR*) families, Ret proto-oncogene (*RET*), protein tyrosine kinase 6 (*BRK*), TEK receptor tyrosine kinase (*TIE2*), and members of the EPH receptor and Src kinase families. These kinases play a role in both normal cellular functions and pathological processes such as oncogenesis, metastasis, tumor angiogenesis, and maintenance of the tumor microenvironment. The *RET* M918T mutation in MTC is classified as Level B based on emerging Phase III clinical data (Figure 3).

Level C is divided into two sub-levels: C1 and C2. Level C1 includes alterations that have received FDA/NMPA approval or are recommended by clinical professional guidelines as predictive of response to therapies in different types of tumors. For example, anaplastic lymphoma kinase (*ALK*) rearrangements are found in 2–7% of non-small-cell lung cancer (NSCLC) patients [12,13]. The FDA has approved several ALK TKIs, including crizotinib, alectinib, ceritinib, brigatinib, and lorlatinib, for treating *ALK*-positive NSCLC. Studies have also shown that ALK TKIs produce significant clinical responses in *ALK*-positive non-NSCLC patients [14–21]. The available evidence indicates that targeted therapy may be beneficial for these patients. As a result, *ALK* rearrangement is classified as Level C1 in non-NSCLC tumors, based on evidence derived from NSCLC. Level C2 includes alterations that predict biomarkers of response to therapies, as established by small retrospective or prospective studies (Phase I or II) in specific types of tumors. Phosphatase and tensin homolog (PTEN) is often deregulated in advanced prostate cancers, leading to the activation of the PI3K-Akt-mTOR pathway and increased cell survival. A Phase II study (NCT00976755) of everolimus, an mTOR inhibitor, was conducted in patients with metastatic castration-resistant prostate cancer. The study found that *PTEN* deletion was associated with higher disease remission rates and improved progression-free survival (PFS) [22]. According to the emerging Phase II clinical data, *PTEN* deletion is classified as a Level C2 mutation in prostate cancer (Figure 3).

Level D is subdivided into Levels D1 and D2. These levels include alterations that are candidate predictive biomarkers of response to therapies based solely on a few reported cases in specific tumors (Level D1) or preclinical studies (Level D2). Futibatinib is a potent inhibitor that targets the FGFR family. It has high affinity for FGFR 1, 2, 3, and 4, with IC_50_ values below 4 nM. Futibatinib forms a covalent bond with FGFR, effectively inhibiting FGFR phosphorylation, disrupting downstream signaling pathways, and reducing the viability of cancer cells harboring FGFR alterations, including fusions/rearrangements, amplifications, and mutations. Clinical observations have shown that patients with gastric cancer featuring *FGFR2* amplification respond to futibatinib treatment [23,24]. Consequently, in the context of gastric cancer, *FGFR2* amplification has been classified as a Level D1 mutation (Figure 3).

OKAIN classifies evidence for resistance into two main levels: R1 and R2. Level R1 includes alterations that have been approved by the FDA/NMPA or recommended by clinical professional guidelines as predictive of resistance to therapies in specific tumors. An example of this is the *PDGFRA* D842V mutation, which has been shown to cause resistance to imatinib in patients with GISTs (Figure 3). Resistance evidence Level R2 is subdivided into R2B, R2C, and R2D, which correspond to sensitivity evidence levels B, C, and D, respectively. This stratification facilitates the precise weighting of clinical evidence.

### 3.2 Examples of weighted analysis interpretation

**Figure 4:**
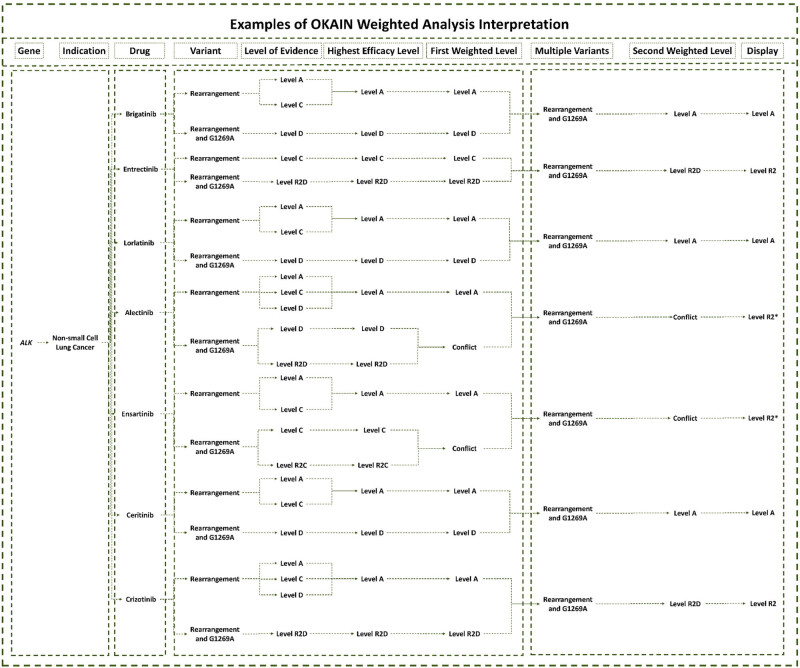
Examples of OKAIN weighted analysis interpretation. The level of evidence shows the efficacy for the specific genomic variant related to a particular drug. The Highest Efficacy Level indicates the maximal therapeutic effect for the specific genomic variant in response to a specific drug. The First Weighted Level indicates the ultimate drug efficacy for the specific genomic variant, as determined after applying a weighting analysis. If the Highest Efficacy Level presents conflicting evidence, the system will output “Conflict.” The Second Weighted Level specifies the definitive efficacy for all variants of the gene. If the highest level presents conflicting evidence, the system outputs “Conflict.” The display shows the ultimate efficacy for all variants of the gene related to the drug. “*” prompting that the sensitivity of *ALK* rearrangement and *ALK* G1269A co-mutation is controversial.

OKAIN logs all clinical data for each genomic alteration, enabling physicians to comprehensively review the therapeutic implications. A single genetic variant may correspond to multiple efficacy levels for a given therapeutic strategy (Figure 4). The evidence analysis system is designed to help physicians obtain conclusive evidence of therapeutic efficacy for cancer patients quickly and succinctly.

The FDA/NMPA has approved several ALK TKIs for treating NSCLC patients with *ALK* rearrangement. Crizotinib, ceritinib, ensartinib, alectinib, brigatinib, and lorlatinib are all designated as Level A for this rearrangement. Additionally, numerous preclinical studies have classified this rearrangement as Level D, predicting sensitivity to ALK TKIs [25–27]. The weighting system prioritizes the highest efficacy level for genomic alterations with multiple levels. Therefore, ALK rearrangement is categorized as Level A for crizotinib, ceritinib, ensartinib, alectinib, brigatinib, and lorlatinib. If conflicting evidence is present for the highest level, the system outputs “Conflict.” As shown in Figure 4, the *ALK* rearrangement and ALK G1269A co-mutation are classified as Level D and R2D for alectinib, according to various literature sources [28–31]. In this case, the system would output “Conflict” when weighted. OKAIN highlights resistant clinical evidence to ensure patients do not lose valuable treatment time due to potentially ineffective targeted drugs, even if the *ALK* rearrangement is classified as Level A for alectinib. The co-occurrence of *ALK* rearrangement and *ALK* G1269A mutation is ultimately categorized as “Level R2*”, with “*” indicating that the sensitivity of this co-mutation to alectinib is controversial. Similarly, *ALK* rearrangement and *ALK* G1269A co-mutations are ultimately classified as “Level R2*” [32,33]. When the highest level of evidence indicates resistance, the *ALK* rearrangement and *ALK* G1269A co-mutations are ultimately classified as Level R2 for crizotinib and entrectinib [25,30,31].

### 3.3 Quantity of evidence in the OKAIN knowledge base

**Figure 5:**
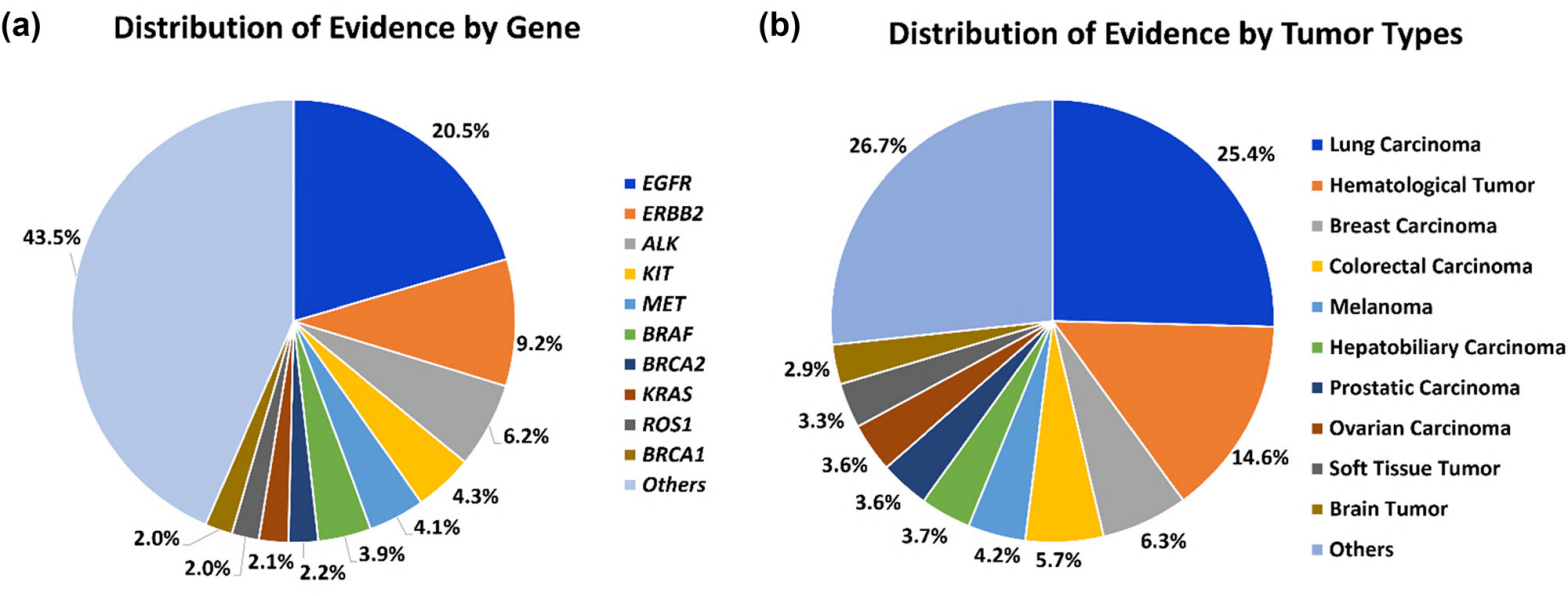
Distribution of therapeutic evidence. (a) Distribution of therapeutic evidence for genes in OKAIN knowledge base when ranked by the number of interpretations in the database. (b) Distribution of therapeutic evidence for different tumor types in OKAIN knowledge base when ranked by the number of interpretations in the clinical data.

To date, OKAIN has amassed over 100,000 variants in 1,239 cancer-associated genes, encompassing 12,409 entries of therapeutic evidence in 471 genes. This collection highlights 2,600 Level A evidence entries in 66 genes, with 864 entries derived from either the NMPA label or Chinese guidelines (Table S1). The *EGFR*, erb-B2 receptor tyrosine kinase 2 (ERBB2/HER2), *ALK*, *KIT*, MET proto-oncogene (*MET*), and B-Raf proto-oncogene (*BRAF*) genes are associated with the largest numbers of therapeutic evidence (Figure 5a). Lung carcinoma is the largest proportion of therapeutic evidence by tumor type, due to its association with the largest number of therapeutic strategies (Figure 5b).

### 3.4 Clinically actionable variations in Chinese patients

**Figure 6:**
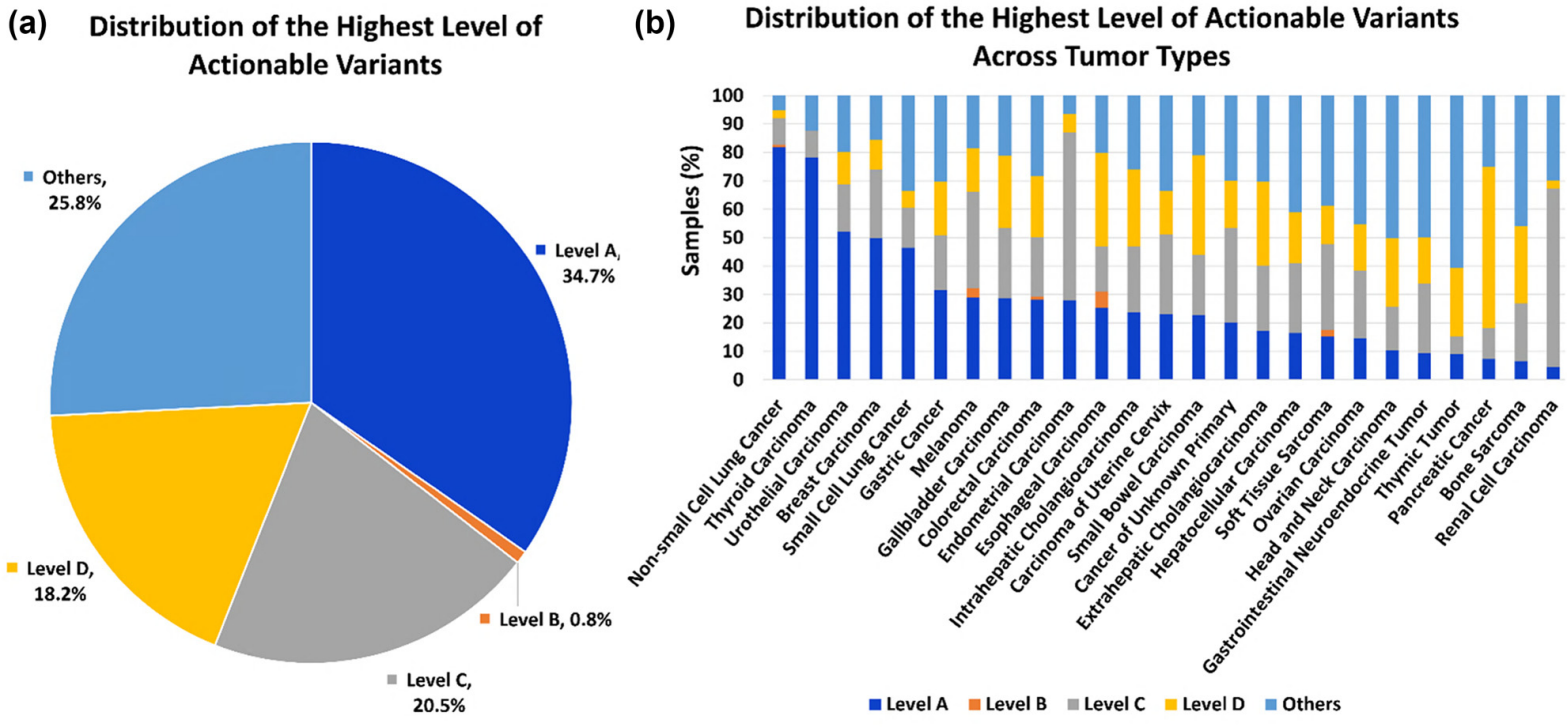
Clinical actionability of somatic variants in the China Pan-cancer cohort. Variants were assigned to different levels of clinical actionability according to OKAIN knowledge base. (a) Distribution of the highest level of actionable variants across all patients. (b) Distribution of highest level of actionable variants across tumor types.

To quantify the potential clinical impact of prospective broad tumor genomic testing, we annotated the variants in a publicly available dataset of 10,194 solid tumors with China Pan-cancer cohort (OrigiMed, Nature 2022) using the OKAIN knowledge base (Version 1.0, 2024) and the OncoKB (http://oncokb.org/, v4.13) knowledge base, respectively [34]. As shown in Figure 6, Tables S2 and S3, the proportions of OKAIN and OncoKB actionability were 74.1% of patients (*N*  =  7,555 harbored at least one gene variant with a variable highest level of clinical evidence, Level A, 34.7%; Level B, 0.8%; Level C, 20.5%; Level D, 18.2%) and 67.8% (*N*  =  6,911, Level 1, 34.5%; Level 2, 1.0%; Level 3, 19.4%; Level 4, 12.9%), respectively. Complete concordance in clinical actionability annotations was observed, with all 6,911 OncoKB-annotated actionable cases similarly classified as actionable by OAKIN. McNemar’s test was used to assess inter-system differences. Relative to OncoKB, the OKAIN knowledge base demonstrated significantly broader patient coverage (74.1 vs 67.8%; *P* < 0.001) for targeted or immunotherapeutic eligibility, reflecting its systematic evidence aggregation framework for genomic aberration-specific efficacy data.

Discrepancies between OncoKB and OKAIN in identifying patients for therapies and drug sensitivities to actionable variants are highlighted by differences in their clinical assessment and evidence integration (Tables S4, S5 and S6). These variations may stem from OKAIN’s inclusion of additional clinical factors, such as hormone receptor (HR) and HER2 status, in its assessment criteria. The FDA’s approval of capivasertib, in combination with fulvestrant for adult patients with HR-positive, HER2-negative locally advanced or metastatic breast cancer with *PIK3CA/AKT1/PTEN*-alterations post-progression on endocrine therapy, underscores the significance of these factors. When a patient’s HR or HER2 status is either unknown or not HR-positive and HER2-negative, the evidence level assigned to capivasertib for treating breast cancer is adjusted to Level C. Additionally, it may stem from the inclusion of drugs approved in China and recommended by the Chinese Society of Clinical Oncology (CSCO) guidelines (https://www.csco.org.cn/) in OKAIN, which are not listed in OncoKB, such as almonertinib. Moreover, OKAIN’s consideration of a broader evidence base may explain these differences. For example, based on clinical trial data, patients with renal cell carcinoma who have Von Hippel-Lindau tumor suppressor (*VHL*) alterations may benefit from treatments like sunitinib, sorafenib, bevacizumab, or axitinib [35]. Concurrently, preclinical findings indicate that GISTs harboring the *KIT* A502_Y503dup mutation may be less responsive to imatinib therapy [36].

## 4 Discussion

The adoption of NGS in clinical genetic testing has become widespread, leading to the discovery of numerous genetic variants that may impact clinical decision-making. This highlights the significance and advantages of standardized interpretation of these variants [9]. There are several resources available to aid in the clinical interpretation of genetic alterations, such as CIViC [4], OncoKB [5], COSMIC [37], the JAX Clinical Knowledge base [38], the Cancer Driver Log [39], and the Precision Medicine Knowledge Base [40]. These databases offer either the final clinical annotation results or multiple pieces of clinical interpretation evidence related to the alterations. However, until now, there has been a lack of databases published specifically to interpret genetic variants in China. In this context, OKAIN emerges as the first precision oncology knowledge base published in China. This resource offers physicians detailed and structured interpretations of cancer mutations found in patient tumors, along with evidence of therapeutic efficacy through a weighted analysis system. Its potential to enhance precision oncology practice in China is significant.

Like other classification systems [5,9], OKAIN divides the sensitivity evidence into four levels for each mutation, based on the available clinical and preclinical data that support the use of the mutation as a predictive biomarker. The evidence sensitivity levels, ranked from highest to lowest, are A, B, C, and D. Therapeutic implications approved by FDA/NMPA or recommended by clinical professional guidelines for specific tumors are classified as level A. All other therapeutic implications are classified as levels B, C, and D, respectively. The significance of the latter lies in its potential to offer new therapeutic strategies for cancer patients, including off-label use of cancer drugs and enrollment in appropriate clinical trials. However, we do not recommend using cancer drugs off-label for different tumor types due to the limited evidence supporting their efficacy. It is important to consider the safety profile and accessibility of these medications as well as the variability in their effectiveness across different tumors. For example, the results of a phase II trial (NCT00903175) demonstrate the variability of patient outcomes when treated with everolimus for renal cell carcinoma. Patients who retained PTEN expression had a median PFS of 5.3 months, while those who lost PTEN expression experienced a significantly longer median PFS of 10.5 months (*P* < 0.001) [41]. This finding suggests that PTEN status may have predictive value in this context. In contrast, data from another study on transitional cell carcinoma suggest that PTEN loss may be associated with resistance to everolimus. This indicates that the efficacy of this drug is not consistent across different tumor types and that molecular profiles should be carefully considered before off-label application [42].

Although clinical and preclinical studies suggest that certain gene mutations may respond to specific targeted drugs, it is important to note that the gene may not actually be the target of the drug. Currently, there is insufficient research to explain this phenomenon adequately. For example, biomarker analyses of the BERIL-1 trial showed that patients with squamous cell carcinoma of the head and neck, who also had TP53 alterations, benefited from the combination of buparlisib and paclitaxel in terms of survival [43]. However, further investigation is required to establish a definitive link between TP53 alterations and improved outcomes with this drug combination. Therefore, although TP*53* alterations are not currently considered agents in OKAIN, they are still recorded for reference.

OKAIN continuously records new clinical data, including updates to NMPA/FDA labels, professional guidelines, and scientific literature, ensuring that the weighted level of evidence remains current. For example, *RET* rearrangement was not considered as level A in thyroid cancer until December 1, 2020, when the FDA approved pralsetinib for the treatment of adult and pediatric patients (12 years of age and older) with advanced or metastatic *RET* fusion-positive thyroid cancer who require systemic therapy and are radioactive iodine-refractory. This timely inclusion of new clinical data enhances the utility of OKAIN for making informed therapeutic decisions.

OKAIN plans to conduct weighted analyses for complex variants, including multiple genetic variants, rather than solely focusing on single-gene alterations. Additionally, they aim to establish a platform where physicians can engage in discussions and provide comments on variant annotations, aiding in their accurate understanding of these alterations.

OKAIN has entered clinical practice across multiple settings, supporting critical oncology workflows including: (1) interpretation of NGS reports to deliver clinically actionable results, (2) genetic counseling sessions, and (3) Molecular Tumor Board discussions by providing evidence for therapeutic decision-making. Ongoing real-world studies are quantitatively assessing OKAIN’s implementation fidelity, impact on therapeutic recommendations, and broader utility in precision oncology pathways.

## 5 Conclusions

OKAIN is a knowledge base for cancer genomics and precision treatment, including therapies approved in China and those recommended by Chinese clinical guidelines. OKAIN presents an extensive array of evidence, enhanced by a specialized weighting system, facilitating precision treatment for clinicians and patients with complex variations. Analyzing patient variants with OKAIN reveals more actionable targeted therapy or immunotherapy options, potentially improving treatment outcomes.

## Supplementary Material

Supplementary Table

## References

[1] Schwartzberg L, Kim ES, Liu D, Schrag D. Precision oncology: who, how, what, when, and when not? Am Soc Clin Oncol Educ Book. 2017;7:160–9. 10.1200/EDBK_174176 28561651

[2] Li X, Warner JL. A review of precision oncology knowledge bases for determining the clinical actionability of genetic variants. Front Cell Dev Biol. 2020;8:48. 10.3389/fcell.2020.00048 PMC7026022 32117976

[3] Sicklick JK, Kato S, Okamura R, Schwaederle M, Hahn ME, Williams CB, et al. Molecular profiling of cancer patients enables personalized combination therapy: the I-PREDICT study. Nat Med. 2019;25(5):744–50. 10.1038/s41591-019-0407-5 PMC6553618 31011206

[4] Griffith M, Spies NC, Krysiak K, McMichael JF, Coffman AC, Danos AM, et al. CIViC is a community knowledge base for expert crowdsourcing the clinical interpretation of variants in cancer. Nat Genet. 2017;49(2):170–4. 10.1038/ng.3774 PMC5367263 28138153

[5] Chakravarty D, Gao J, Phillips SM, Phillips SM, Kundra R, Zhang H, et al. OncoKB: a precision oncology knowledge base. JCO Precis Oncol. 2017;1:1–16. 10.1200/PO.17.00011 PMC5586540 28890946

[6] Gray SW, Hicks-Courant K, Cronin A, Rollins BJ, Weeks JC. Physicians’ attitudes about multiplex tumor genomic testing. J Clin Oncol. 2014;32(13):1317–23. 10.1200/JCO.2013.52.4298 PMC3992721 24663044

[7] Den Dunnen JT, Dalgleish R, Maglott DR, Hart RK, Greenblatt MS, McGowan-Jordan J, et al. HGVS recommendations for the description of sequence variants: 2016 update. Hum Mutat. 2016;37(6):564–9. 10.1002/humu.22981 26931183

[8] Den Dunnen JT, Antonarakis SE. Mutation nomenclature extensions and suggestions to describe complex mutations: a discussion. Hum Mutat. 2000;15(1):7–12. 10.1002/(SICI)1098-1004(200001)15:1<7::AID-HUMU4>3.0.CO;2-N 10612815

[9] Li MM, Datto M, Duncavage EJ, Kulkarni S, Lindeman NI, Roy S, et al. Standards and guidelines for the interpretation and reporting of sequence variants in cancer: a joint consensus recommendation of the Association for Molecular Pathology, American Society of Clinical Oncology, and College of American Pathologists. J Mol Diagn. 2017;19(1):4–23. 10.1016/j.jmoldx.2016.10.002 PMC5707196 27993330

[10] Mateo J, Chakravarty D, Dienstmann R, Jezdic S, Gonzalez-Perez A, Lopez-Bigas N, et al. A framework to rank genomic alterations as targets for cancer precision medicine: the esmo scale for clinical actionability of molecular targets (ESCAT). Ann Oncol. 2018;29(9):1895–902. 10.1093/annonc/mdy263 PMC6158764 30137196

[11] Langmuir PB, Yver A. Vandetanib for the treatment of thyroid cancer. Clin Pharmacol Ther. 2012;91(1):71–80. 10.1038/clpt.2011.272 22158569

[12] Soda M, Choi YL, Enomoto M, Takada S, Yamashita Y, Ishikawa S, et al. Identification of the transforming EML4-ALK fusion gene in non-small-cell lung cancer. Nature. 2007;448(7153):561–6. 10.1038/nature05945 17625570

[13] Kwak EL, Bang YJ, Camidge DR, Shaw AT, Solomon B, Maki RG, et al. Anaplastic lymphoma kinase inhibition in non-small-cell lung cancer. N Engl J Med. 2010;363(18):1693–703. 10.1056/NEJMoa1006448 PMC3014291 20979469

[14] Hsiao SY, He HL, Weng TS, Lin CY, Chao CM, Huang WT, et al. Colorectal cancer with EML4-ALK fusion gene response to alectinib: a case report and review of the literature. Case Rep Oncol. 2021;14(1):232–8. 10.1159/000511069 PMC7983623 33776709

[15] Hu J, Zhang B, Yao F, Fu Y, Chen D, Li D, et al. Acquired multiple mutations ALK I1171N, L1196M and G1202R mediate lorlatinib resistance in EML4-ALK-rearranged malignant pleural mesothelioma: a case report. Ther Adv Respir Dis. 2020;14:1753466620935770. 10.1177/1753466620935770 PMC7328355 32600123

[16] Godbert Y, de Figueiredo BH, Bonichon F, Chibon F, Hostein I, Pérot G, et al. Remarkable response to crizotinib in woman with anaplastic lymphoma kinase-rearranged anaplastic thyroid carcinoma. J Clin Oncol. 2015;33(20):e84–7. 10.1200/JCO.2013.49.6596 24687827

[17] Zhou Y, Lizaso A, Mao X, Yang N, Zhang Y. Novel AMBRA1-ALK fusion identified by next-generation sequencing in advanced gallbladder cancer responds to crizotinib: a case report. Ann Transl Med. 2020;8(17):1099. 10.21037/atm-20-1007 PMC7575933 33145318

[18] Hui B, Zhang J, Shi X, Xing F, Shao YW, Wang Y, et al. EML4-ALK, a potential therapeutic target that responds to alectinib in ovarian cancer. Jpn J Clin Oncol. 2020;50(12):1470–4. 10.1093/jjco/hyaa156 32845005

[19] Zhao P, Peng L, Wu W, Zheng Y, Jiang W, Zhang H, et al. Carcinoma of unknown primary with EML4-ALK fusion response to ALK inhibitors. Oncologist. 2019;24(4):449–54. 10.1634/theoncologist.2018-0439 PMC6459257 30679319

[20] Yakirevich E, Resnick MB, Mangray S, Wheeler M, Jackson CL, Lombardo KA, et al. Oncogenic ALK fusion in rare and aggressive subtype of colorectal adenocarcinoma as a potential therapeutic target. Clin Cancer Res. 2016;22(15):3831–40. 10.1158/1078-0432.CCR-15-3000 26933125

[21] Amatu A, Somaschini A, Cerea G, Bosotti R, Valtorta E, Buonandi P, et al. Novel CAD-ALK gene rearrangement is drugable by entrectinib in colorectal cancer. Br J Cancer. 2015;113(12):1730–4. 10.1038/bjc.2015.401 PMC4701996 26633560

[22] Templeton AJ, Dutoit V, Cathomas R, Rothermundt C, Bärtschi D, Dröge C, et al. Phase 2 trial of single-agent everolimus in chemotherapy-naive patients with castration-resistant prostate cancer (SAKK 08/08). Eur Urol. 2013;64(1):150–8. 10.1016/j.eururo.2013.03.040 23582881

[23] Doi T, Shitara K, Kojima T, Kuboki Y, Matsubara N, Bando H, et al. Phase I study of the irreversible fibroblast growth factor receptor 1–4 inhibitor futibatinib in Japanese patients with advanced solid tumors. Cancer Sci. 2023;114(2):574. 10.1111/cas.15486 PMC9899610 35838190

[24] Meric-Bernstam F, Bahleda R, Hierro C, Sanson M, Bridgewater J, Arkenau H, et al. Futibatinib, an irreversible FGFR1–4 inhibitor, in patients with advanced solid tumors harboring FGF/FGFR aberrations: a phase I dose-expansion study. Cancer Discovery. 2022;12(2):402–15. 10.1158/2159-8290.CD-21-0697 PMC9762334 34551969

[25] Mizuta H, Okada K, Araki M, Adachi J, Takemoto A, Kutkowska J, et al. Gilteritinib overcomes lorlatinib resistance in ALK-rearranged cancer. Nat Commun. 2021;12(1):1261. 10.1038/s41467-021-21396-w PMC7904790 33627640

[26] Mologni L, Ceccon M, Pirola A, Chiriano G, Piazza R, Scapozza L, et al. NPM/ALK mutants resistant to ASP3026 display variable sensitivity to alternative ALK inhibitors but succumb to the novel compound PF-06463922. Oncotarget. 2015;6(8):5720–34. 10.18632/oncotarget.3122 PMC4467397 25749034

[27] Gainor JF, Dardaei L, Yoda S, Friboulet L, Leshchiner I, Katayama R, et al. Molecular mechanisms of resistance to first-and second-generation ALK inhibitors in ALK-rearranged lung cancer. Cancer Discovery. 2016;6(10):1118–33. 10.1158/2159-8290.CD-16-0596 PMC5050111 27432227

[28] Yoshimura Y, Kurasawa M, Yorozu K, Puig O, Bordogna W, Harada N. Antitumor activity of alectinib, a selective ALK inhibitor, in an ALK-positive NSCLC cell line harboring G1269A mutation: efficacy of alectinib against ALK G1269A mutated cells. Cancer Chemother Pharmacol. 2016;77(3):623–8. 10.1007/s00280-016-2977-y 26849637

[29] Kim S, Kim TM, Kim DW, Go H, Keam B, Lee SH, et al. Heterogeneity of genetic changes associated with acquired crizotinib resistance in ALK-rearranged lung cancer. J Thorac Oncol. 2013;8(4):415–22. 10.1097/JTO.0b013e318283dcc0 23344087

[30] Fontana D, Ceccon M, Gambacorti-Passerini C, Mologni L. Activity of second-generation ALK inhibitors against crizotinib-resistant mutants in an NPM-ALK model compared to EML4-ALK. Cancer Med. 2015;4(7):953–65. 10.1002/cam4.413 PMC4529334 25727400

[31] Amin AD, Li L, Rajan SS, Gokhale V, Groysman MJ, Pongtornpipat P, et al. TKI sensitivity patterns of novel kinase-domain mutations suggest therapeutic opportunities for patients with resistant ALK + tumors. Oncotarget. 2016;7(17):23715–29. 10.18632/oncotarget.8173 PMC5029658 27009859

[32] Yang Y, Huang J, Wang T, Zhou J, Zheng J, Feng J, et al. Decoding the evolutionary response to ensartinib in patients with ALK-positive NSCLC by dynamic circulating tumor DNA sequencing. J Thorac Oncol. 2021;16(5):827–39. 10.1016/j.jtho.2021.01.1615 33588113

[33] Yang Y, Zhou J, Zhou J, Feng J, Zhuang W, Chen J, et al. Efficacy, safety, and biomarker analysis of ensartinib in crizotinib-resistant, ALK-positive non-small-cell lung cancer: a multicentre, phase 2 trial. Lancet Respir Med. 2020;8(1):45–53. 10.1016/S2213-2600(19)30252-8 31628085

[34] Wu L, Yao H, Chen H, Wang A, Guo K, Gou W, et al. Landscape of somatic alterations in large-scale solid tumors from an Asian population. Nat Commun. 2022;13(1):4264. 10.1038/s41467-022-31780-9 PMC9308789 35871175

[35] Choueiri TK, Vaziri SA, Jaeger E, Elson P, Wood L, Bhalla IP, et al. von Hippel-Lindau gene status and response to vascular endothelial growth factor targeted therapy for metastatic clear cell renal cell carcinoma. J Urol. 2008;180(3):860–6. 10.1016/j.juro.2008.05.015 18635227

[36] Smith BD, Kaufman MD, Lu WP, Gupta A, Leary CB, Wise SC, et al. Ripretinib (DCC-2618) is a switch control kinase inhibitor of a broad spectrum of oncogenic and drug-resistant KIT and PDGFRA variants. Cancer Cell. 2019;35(5):738–51. 10.1016/j.ccell.2019.04.006 31085175

[37] Forbes SA, Beare D, Boutselakis H, Bamford S, Bindal N, Tate J, et al. COSMIC: somatic cancer genetics at high-resolution. Nucleic Acids Res. 2017;45(D1):D777–83. 10.1093/nar/gkw1121 PMC5210583 27899578

[38] Patterson SE, Liu R, Statz CM, Durkin D, Lakshminarayana A, Mockus SM. The clinical trial landscape in oncology and connectivity of somatic mutational profiles to targeted therapies. Hum Genomics. 2016;10:4. 10.1186/s40246-016-0061-7 PMC4715272 26772741

[39] Damodaran S, Miya J, Kautto E, Zhu E, Samorodnitsky E, Datta J, et al. Cancer driver log (CanDL): catalog of potentially actionable cancer mutations. J Mol Diagn. 2015;17(5):554–9. 10.1016/j.jmoldx.2015.05.002 PMC4597274 26320871

[40] Huang L, Fernandes H, Zia H, Tavassoli P, Rennert H, Pisapia D, et al. The cancer precision medicine knowledge base for structured clinical-grade mutations and interpretations. J Am Med Inf Assoc. 2017;24(3):513–9. 10.1093/jamia/ocw148 PMC5391733 27789569

[41] Voss MH, Chen D, Reising A, Marker M, Shi J, Xu J, et al. PTEN expression, not mutation status in TSC1, TSC2, or mTOR, correlates with the outcome on everolimus in patients with renal cell carcinoma treated on the randomized RECORD-3 trial. Clin Cancer Res. 2019;25(2):506–14. 10.1158/1078-0432.CCR-18-1833 30327302

[42] Seront E, Rottey S, Sautois B, Kerger J, D’Hondt LA, Verschaeve V, et al. Phase II study of everolimus in patients with locally advanced or metastatic transitional cell carcinoma of the urothelial tract: clinical activity, molecular response, and biomarkers. Ann Oncol. 2012;23(10):2663–70. 10.1093/annonc/mds057 22473592

[43] Soulieres D, Licitra L, Mesía R, Remenár E, Li S, Karpenko A, et al. Molecular alterations and buparlisib efficacy in patients with squamous cell carcinoma of the head and neck: biomarker analysis from BERIL-1. Clin Cancer Res. 2018;24(11):2505–16. 10.1158/1078-0432.CCR-17-2644 29490986

