# Supplementary material for: OKAIN: A comprehensive oncology knowledge base for the interpretation of clinically actionable alterations
Source: Open Med (Wars). 2025 Oct 30;20(1):20251289. doi: 10.1515/med-2025-1289 (PMC12596872; doi:10.1515/med-2025-1289)
Supplement: Supplementary Table [file med-2025-1289-sm.pdf]

Supplementary material

Table S1: Distribution of therapeutic evidence in the OKAIN knowledge base

Interpretation metric	No. of genes	No. of alterations
Total	471	12,409
Level A	66	2,600
Level B	31	180
Level C	81	676
Level D	188	5,562
Level R1	12	136
Level R2	110	3,255

Table S2: Clinical actionability of somatic variants in the China pan-cancer cohort based on the OKAIN knowledge base

	Level A (%)	Level B (%)	Level C (%)	Level D (%)	Others (%)
Bone sarcoma	6.6	0.0	20.2	27.3	45.9
Breast carcinoma	49.8	0.0	24.1	10.5	15.6
Cancer of unknown primary	20.0	0.0	33.3	16.7	30.0
Carcinoma of uterine cervix	23.1	0.0	27.9	15.4	33.6
Colorectal carcinoma	28.1	1.2	20.7	21.7	28.3
Esophageal carcinoma	25.2	5.7	15.9	33.0	20.2
Extrahepatic cholangiocarcinoma	17.1	0.0	23.1	29.6	30.2
Gallbladder carcinoma	28.8	0.0	24.6	25.4	21.2
Gastric cancer	31.5	0.0	19.4	18.7	30.4
Gastrointestinal neuroendocrine tumor	9.5	0.0	24.3	16.2	50.0
Head and neck carcinoma	10.3	0.0	15.4	24.0	50.3
Intrahepatic cholangiocarcinoma	23.6	0.0	23.4	27.0	26.0
Renal cell carcinoma	4.5	0.0	62.7	2.9	29.9
Hepatocellular carcinoma	16.4	0.0	24.6	17.7	41.3
Melanoma	28.8	3.4	33.9	15.3	18.6
Non-small cell lung cancer	81.9	0.6	9.5	2.7	5.3
Ovarian carcinoma	14.6	0.0	23.8	16.5	45.1
Pancreatic cancer	7.2	0.0	10.8	56.8	25.2
Small bowel carcinoma	22.8	0.0	21.1	35.1	21.0
Small cell lung cancer	46.4	0.0	14.1	5.9	33.6
Soft tissue sarcoma	15.2	2.3	30.1	13.5	38.9
Thymic tumor	9.1	0.0	6.1	24.2	60.6
Thyroid carcinoma	78.1	0.0	9.4	0.0	12.5
Urothelial carcinoma	52.1	0.0	16.7	11.5	19.7
Endometrial carcinoma	27.9	0.0	59.0	6.6	6.5
All solid tumors	**34.7**	**0.8**	**20.5**	**18.2**	**25.8**

Table S3: Clinical actionability of somatic variants in the China pan-cancer cohort based on the OncoKB knowledge base

	**Level 1 (%)**	**Level 2 (%)**	**Level 3 (%)**	**Level 4 (%)**	**Others (%)**
Bone sarcoma	4.4	2.2	10.9	9.3	73.2
Breast carcinoma	68.7	0.0	5.0	1.9	24.4
Cancer of unknown primary	20.0	0.0	32.5	13.3	34.2
Carcinoma of uterine cervix	20.2	0.0	30.8	3.8	45.2
Colorectal carcinoma	26.6	1.7	19.9	25.5	26.3
Esophageal carcinoma	27.7	0.0	14.4	19.2	38.7
Extrahepatic cholangiocarcinoma	15.7	1.4	19.4	33.6	29.9
Gallbladder carcinoma	17.9	10.8	24.6	17.1	29.6
Gastric cancer	31.5	0.0	18.6	10.2	39.7
Gastrointestinal neuroendocrine tumor	8.1	0.0	21.6	14.9	55.4
Head and neck carcinoma	9.7	0.0	16.6	13.7	60.0
Intrahepatic cholangiocarcinoma	22.5	1.1	21.3	28.8	26.3
Renal cell carcinoma	4.5	0.0	14.3	5.5	75.7
Hepatocellular carcinoma	16.4	0.0	21.7	10.6	51.3
Melanoma	20.3	1.7	39.0	10.2	28.8
Non-small cell lung cancer	81.5	0.6	6.8	4.1	7.0
Ovarian carcinoma	14.6	0.0	22.2	8.4	54.8
Pancreatic cancer	4.2	3.0	72.5	11.0	9.3
Small bowel carcinoma	22.8	0.0	21.1	43.9	12.2
Small cell lung cancer	46.4	0.0	13.2	0.5	39.9
Soft tissue sarcoma	16.5	1.4	19.1	9.3	53.7
Thymic tumor	9.1	0.0	6.1	24.2	60.6
Thyroid carcinoma	78.1	0.0	9.4	0.0	12.5
Urothelial carcinoma	47.9	0.0	25.0	9.4	17.7
Endometrial carcinoma	24.6	1.6	60.7	6.6	6.5
All solid tumors	**34.5**	**1.0**	**19.4**	**12.9**	**32.2**

Table S4: Population of patients harboring specific biomarkers who may benefit from standard therapies based on the OKAIN knowledge base

	TMB – H	*EGFR*	*ERBB2*	*MSI – H*	*ALK*	*KRAS*	*BRAF*	*NTRK3*	*RET*	*MET*	*IDH1*	*bKIT*	*ROS1*	*PIK3CA*	*FGFR2*	*BRCA2*	*BRCA1*	*NTRK1*	*FGFR3*	*PDGFRA*	*PALB2*	*ESR1*	*PTEN*	*NTRK2*	*AKT1*	*NRAS*
Non-small cell lung cancer	544	909	71	3	127	88	19	8	36	48			36					3								
Colorectal carcinoma	256		22	102		28	54	4	2									5								
Gastric cancer	222		69	49			2	7	1									2						1		
Liver hepatocellular carcinoma	179						2	3	1									1								
Esophageal carcinoma	150		1					3																		
Small cell lung cancer	102							4																		
Intrahepatic cholangiocarcinoma	55		9	13			6	1	1		36				28			1								
Breast carcinoma	47		82					3	2					34		13	7					3	3		1	
Extrahepatic cholangiocarcinoma	43		8	5			2	3			4				5											
Gallbladder carcinoma	40		33				1	2																		
Urothelial carcinoma	38		8	2				1											17							
Cancer of unknown primary	22						1	1																		
Carcinoma of uterine cervix	21		3																							
Soft tissue sarcoma	21			1			2	10				37				2		9		6				1		
Ovarian carcinoma	20			2				3								5	11									
Pancreatic cancer	19			2		4	1	2								6	3				4					
Head and neck carcinoma	15		2	1				2																		
Uterine corpus endometrial carcinoma	15		2	2																						
Renal cell carcinoma	14																									
Small bowel carcinoma	12			3				1																		
Gastrointestinal neuroendocrine tumor	6		1																							
Melanoma	5						10		1			2													1	
Bone sarcoma	4			1				4			4															
Thymic tumor	3																									
Thyroid carcinoma	1						15	1	9																	
All solid tumors	**1854**	**909**	**311**	**186**	**127**	**120**	**115**	**63**	**53**	**48**	**44**	**39**	**36**	**34**	**33**	**26**	**21**	**21**	**17**	**6**	**4**	**3**	**3**	**2**	**1**	**1**

Table S5: Population of patients harboring specific biomarkers who may benefit from standard therapies based on the OncoKB knowledge base

	TMB − H	*EGFR*	*ERBB2*	*MSI − H*	*ALK*	*KRAS*	*PIK3CA*	*BRAF*	*NTRK3*	*RET*	*KIT*	*MET*	*IDH1*	*ROS1*	*FGFR2*	*PTEN*	*NTRK1*	*FGFR3*	*AKT1*	*BRCA1*	*BRCA2*	*ESR1*	*PDGFRA*	*PALB2*	*SMARCB1*	*NTRK2*
Non-small cell lung cancer	544	912	72	3	127	88		19	8	36		49		36			3									
Colorectal carcinoma	256		27	102		28		54	4	2							5									
Gastric cancer	222		69	49				2	7	1							2									1
Liver hepatocellular carcinoma	179							2	3	1							1									
Esophageal carcinoma	150		18						3																	
Small cell lung cancer	102								4																	
Intrahepatic cholangiocarcinoma	55		9	13				6	1	1			36		28		1									
Breast carcinoma	47		77				116		3	2						31			15			7				
Extrahepatic cholangiocarcinoma	43		8	5				2	3				4		5											
Gallbladder carcinoma	40		33					1	2																	
Urothelial carcinoma	38			2					1									16								
Cancer of unknown primary	22							1	1																	
Carcinoma of uterine cervix	21																									
Soft tissue sarcoma	21			1				2	10		49						9				2		7		3	1
Ovarian carcinoma	20			2					3											11	5					
Pancreatic cancer	19			2		4		1	2											3	6			4		
Head and neck carcinoma	15			1					2																	
Uterine corpus endometrial carcinoma	15		1	2																						
Renal cell carcinoma	14																									
Small bowel carcinoma	12			3					1																	
Gastrointestinal neuroendocrine tumor	6																									

(*Continued*)

Table S5: *Continued*

	TMB − H	*EGFR*	*ERBB2*	*MSI − H*	*ALK*	*KRAS*	*PIK3CA*	*BRAF*	*NTRK3*	*RET*	*KIT*	*MET*	*IDH1*	*ROS1*	*FGFR2*	*PTEN*	*NTRK1*	*FGFR3*	*AKT1*	*BRCA1*	*BRCA2*	*ESR1*	*PDGFRA*	*PALB2*	*SMARCB1*	*NTRK2*
Melanoma	5							8		1	1															
Bone sarcoma	4			1					4				4													
Thymic tumor	3																									
Thyroid carcinoma	1							15	1	9																
All solid tumors	**1854**	**912**	**314**	**186**	**127**	**120**	**116**	**113**	**63**	**53**	**50**	**49**	**44**	**36**	**33**	**31**	**21**	**16**	**15**	**14**	**13**	**7**	**7**	**4**	**3**	**2**

Note: Standard therapies refer to treatments for which OKAIN assigns level A evidence rating. The numbers represent the population of samples per tumor type that harbor all mutations in each biomarker, as analyzed based on the OKAIN knowledge base.

Note: Standard therapies refer to treatments for which OncoKB designates as levels 1 and 2. The numbers represent the population of samples per tumor type that harbor all mutations in each biomarker, as analyzed based on the OncoKB knowledge base.

Table S6: Clinically actionable variants in various tumor types and their potential sensitive drugs

Gene	Variation	Tumor	Drugs recorded in OKAIN or OncoKB			Standard treatment
			**OKAIN and OncoKB**	**OKAIN Only**	**OncoKB Only**	
AKT1	E17K	Breast carcinoma	Capivasertib	—	—	Y
AKT1	E17K	Uterine corpus endometrial carcinoma	Capivasertib	—	—	N
ALK	Fusions	Non-small cell lung cancer	Alectinib, brigatinib, ceritinib, crizotinib, lorlatinib	Ensartinib, iruplinalkib	—	Y
ALK	Fusions	Colorectal carcinoma	Alectinib, brigatinib, ceritinib, crizotinib, lorlatinib	Ensartinib, iruplinalkib	—	N
ARAF	S214C	Non-small cell lung cancer	Sorafenib	Trametinib	—	N
ARID1A	Truncating mutations	Liver hepatocellular carcinoma	Tazemetostat	Dasatinib	—	N
ATM	Oncogenic mutations	Gastric cancer	Olaparib, Talazoparib	—	—	N
BARD1	Oncogenic mutations	Melanoma	Olaparib	—	—	N
BRAF	V600E	Colorectal carcinoma	Encorafenib+Panitumumab, Cetuximab+Encorafenib	—	—	Y
BRAF	Fusions	Colorectal carcinoma	Selumetinib	—	—	N
EGFR	L858R	Non-small cell lung cancer	Afatinib, Dacomitinib, Erlotinib, Erlotinib+Ramucirumab, Gefitinib, Osimertinib	Bevacizumab+Erlotinib, Icotinib, Alflutinib, Befotertinib, Almonertinib	—	Y
EGFR	Exon19 deletion	Non-small cell lung cancer	Afatinib, Dacomitinib, Erlotinib, Erlotinib+Ramucirumab, Gefitinib, Osimertinib	Bevacizumab+Erlotinib, icotinib, alflutinib, befotertinib, almonertinib	—	Y
KRAS	G12C	Pancreatic cancer	Adagrasib, sotorasib	—	—	Y
KRAS	G12C	Colorectal carcinoma	Adagrasib+Panitumumab, Adagrasib+Cetuximab, Panitumumab+Sotorasib, Cetuximab+Sotorasib	Adagrasib, sotorasib	—	Y
KRAS	G12C	Intrahepatic cholangiocarcinoma	Adagrasib+Panitumumab, Adagrasib+Cetuximab, Panitumumab+Sotorasib, Cetuximab+Sotorasib, Adagrasib, Sotorasib	—	—	N
KIT	A502_Y503dup	Gastrointestinal stromal tumor	Regorafenib, ripretinib, sunitinib	—	Imatinib	Y
MSI-H	MSI-H	Colorectal carcinoma	Nivolumab, Nivolumab+Ipilimumab, Pembrolizumab	Dostarlimab-gxly, envafolimab, serplulimab, pucotenlimab, tislelizumab	—	Y

(*Continued*)

Table S6: *Continued*

Gene	Variation	Tumor	Drugs recorded in OKAIN or OncoKB			Standard treatment
			OKAIN and OncoKB	OKAIN Only	OncoKB Only	
PIK3CA	Oncogenic mutations	Colorectal carcinoma	Alpelisib, capivasertib	—	—	N
TMB-H	TMB-H	Non-small cell lung cancer	Pembrolizumab	—	—	Y
TSC1	Oncogenic mutations	Renal cell carcinoma	Everolimus	Temsirolimus, sirolimus	—	N
VHL	Oncogenic mutations	Renal cell carcinoma	—	Belzutifan, sorafenib, sunitinib, bevacizumab, axitinib	—	N

Note: '*Y*' represents that the drugs are considered standard therapy for the actionable variants in the corresponding tumor types, with a level A designation in OKAIN and levels 1 and 2 in OncoKB. '*N*' indicates that the drugs are not standard therapy for the actionable variants in the respective tumor types.
